# Operationalizing preventive care: the preventive health logistics ecosystem framework for aging societies

**DOI:** 10.3389/fpubh.2025.1740511

**Published:** 2025-12-15

**Authors:** Hao Yi Tan, Elizabeth Skylar Loo, Chuan De Foo, Huay Ling Tay, Ken Wah Teo, Lian Leng Low

**Affiliations:** 1 Division of Population Health and Integrated Care, Singapore General Hospital, Singapore, Singapore; 2 Saw Swee Hock School of Public Health, National University of Singapore, Singapore, Singapore; 3 Health Systems Group, Harvard T.H. Chan School of Public Health, Boston, MA, United States; 4 Duke-NUS Medical School, Singapore, Singapore; 5 Singapore University of Social Sciences, Singapore, Singapore; 6 Centre for Population Health Research and Implementation, SingHealth, Singapore, Singapore; 7 Signature Research Programme in Health Services Research and Population Health, Duke-NUS Medical School, Singapore, Singapore; 8 SingHealth Office of Regional Health, Singapore, Singapore

**Keywords:** aging population, aging societies, healthcare logistics, healthcare management, Healthier SG, preventive health, preventive health logistics ecosystem

## Introduction

Populations across the globe are aging at an unprecedented pace. By 2050, one in four people (1) in the region is projected to be over 60 years old, creating escalating demands on health and social care systems. In response, governments have increasingly emphasized preventive health and primary care as cornerstones of sustainable health reform, aiming to reduce the burden of chronic disease and promote healthy longevity.

Singapore has long been preparing itself for these shifts through the launch of various primary care reforms, including the recent implementation of Healthier SG (HSG) (2), a national strategy that reorients the health system toward preventive, primary, and community-based care. Central to this strategy is an ecosystem that promotes enrolment with family physicians (3), designed around a vision that encourages healthier lifestyles, proactive screening for common preventable chronic diseases, strengthening of community support networks and leveraging digital health technologies. While priorities represent a critical policy advance (4–6), implementation science in healthcare often neglects the “last-mile” logistics of preventive care. Indeed, in healthcare supply chain literature, logistics is argued to be underdeveloped and underutilized compared to commercial sectors (7), with limited coordination between suppliers and providers (8).

For clarity, we define several key terms we use in this paper. “Preventive care” refers to organized efforts to reduce disease burden through early detection, behavioral interventions, and proactive management of risk factors. Operationalizing said preventive care denotes the translation of these strategies into implementable workflows. We use “logistics ecosystem” to describe the coordinated movement of people, resources and information required to deliver preventive care effectively. Consistent with WHO conventions, “older adults” are defined as persons aged 60 years and above, while “aging societies” refer to population with rising proportions of older adults resulting in increased demand on health and social systems.

Logistics in preventive care encompasses the end-to-end design and management delivery, from forecasting to last-mile execution, including capacity, scheduling, data flows and workforce deployment. Put simply, preventive health strategies must contend not only with what interventions to deliver, but also with how, when, and where they are delivered—and to whom. HSG cultivates the partnership across different levels of care and between private and public healthcare providers, adding a further dimension to be explored (5).

Neglecting these operational considerations risks entrenching inequities and undermining the effectiveness of otherwise well-designed policies. Older adults, for example, often face barriers in mobility (9), digital literacy (10), and continuity of care across the healthcare system. Providers also face business model pressures when policy requirements constrain profit margins, especially when the vast majority of primary care providers enrolled in HSG are private standalone commercial entities (11). Addressing these challenges requires a logistics environment that ensures timely, accessible and integrated preventive interventions. Yet, the “last mile” of preventive care delivery remains under-theorized and insufficiently embedded in health policy design.

This paper introduces the concept of the Preventive Health Logistics Ecosystem (PHLE), a framework for operationalizing preventive care in aging societies. We examine Healthier SG through the PHLE lens, drawing on three crucial institutions. Singapore's national healthcare supply-chain agency, the Agency for Logistics and Procurement Services (ALPS), provides the logistics backbone beyond hospitals, including vendor management and kitting for community nodes. The Agency for Integrated Care's (AIC) integration mandate operates a shared schedule and data backbone through lead social service agencies, pooling resources and aligning incentives. While examples in this paper draw on the roles of three Singapore institutions, these serve as functional archetypes rather than fixed requirements. The PHLE components (decentralized nodes, health triggers, service flows, integration layers, and metrics) can be operationalized by varying combinations of governmental, private, or community actors depending on local governance structures.

PHLE provides a blueprint for how decentralized care nodes, proactive health triggers, and community-integrated logistics can close systemic gaps in service delivery. By situating logistics expertise alongside clinical and policy reforms, the framework aims to render preventive health not only aspirational but actionable, providing a model for resilient, equitable, people-centered health systems. Although grounded in Singapore's context, the PHLE framework is designed to be applicable across aging societies that share common demographic pressures, including rising multimorbidity, constrained primary-care capacity, uneven geographical access, and fragmentation between clinical and social-care providers. These shared system characteristics provide a basis for adapting the framework beyond Singapore.

## Conceptual framework—The preventive health logistics ecosystem

### Theoretical foundations of the PHLE framework

**Figure 1:**
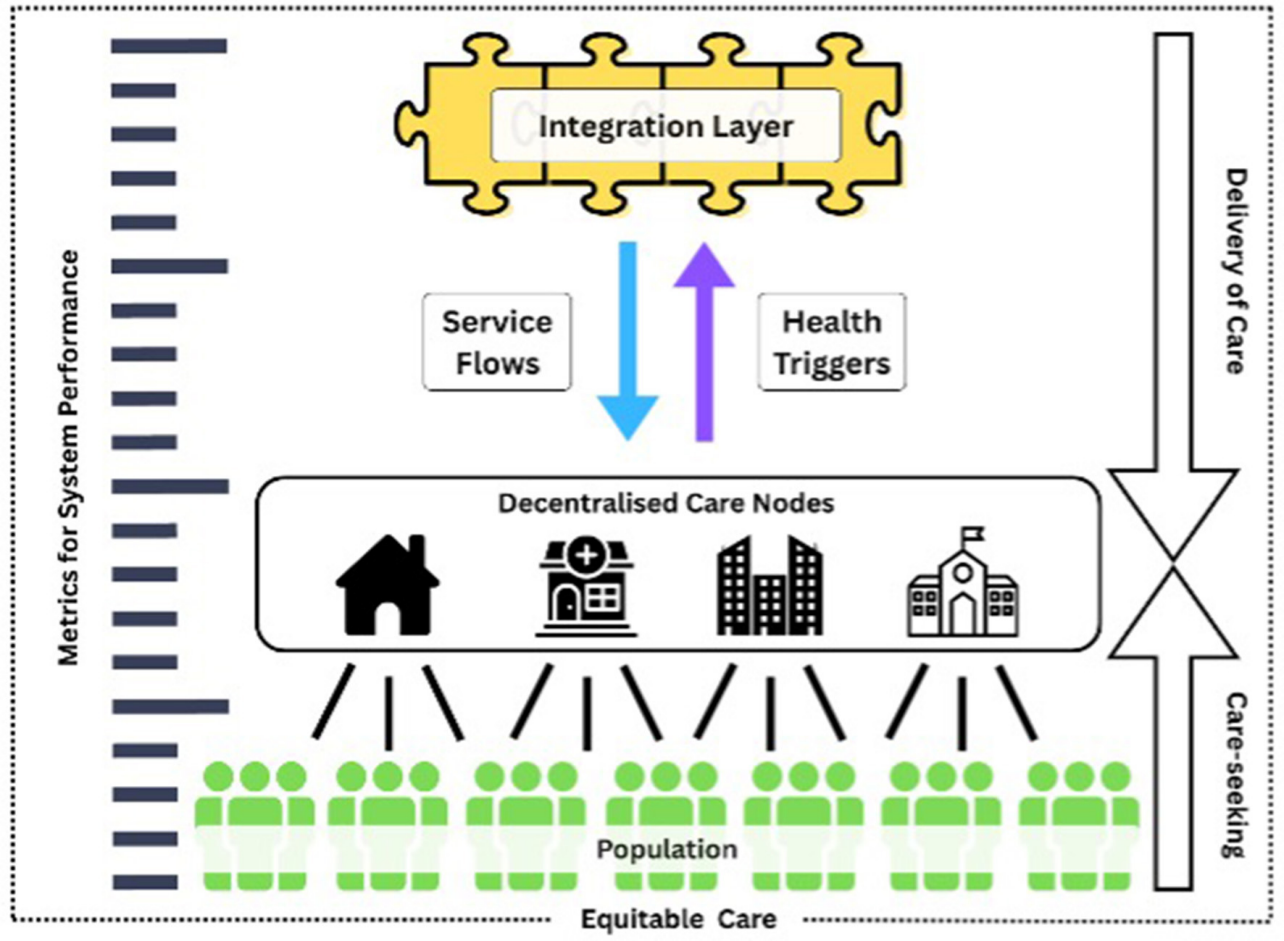
Preventive health logistics ecosystem.

The PHLE framework (see Figure 1) is anchored in an integration of established theories from supply chain management, logistics sciences, and health systems design. At its core, PHLE adapts the Supply Chain Operations Reference (SCOR) model (12), which is widely recognized in commercial sectors for driving end-to-end performance through standardized metrics such as reliability, responsiveness, and flexibility. In this context, SCOR provides a blueprint for orchestrating the entire continuum from risk identification to intervention follow-up, ensuring that each stage is optimally sequenced and resourced. This perspective highlights that preventive interventions require carefully orchestrated flows of people, information, and resources. The framework also leverages queueing theory, particularly Little's Law (13), to address the perennial challenge of service-delivery bottlenecks. By explicitly modeling the relationship between patient arrival rates, service times, and work-in-process, PHLE enables health systems to anticipate and dynamically manage demand surges.

Central to PHLE's design is Donabedian's structure–process–outcome model (14), which provides a systematic lens for linking logistics infrastructure (structure), operational workflows (process), and population health results (outcome). The addition of equity stratification ensures that logistics solutions are not only efficient but also just, prioritizing access for underserved and high-risk groups. This multidimensional theoretical foundation elevates logistics from a technical afterthought to a strategic lever, compelling measurable accountability in preventive health delivery.

### Key components

#### Decentralized care nodes

Decentralized care nodes are designed using principles of network optimisation and the “5 A's” of access (availability, accessibility, accommodation, affordability, and acceptability) (15). By bringing preventive services closer to where people live, these nodes reduce reliance on centralized facilities, shorten the time from risk identification to first contact, and improve equity across neighborhoods. Convenience and proximity are critical determinants of uptake, particularly among older adults and disadvantaged groups, making decentralized nodes essential to ensuring that preventive interventions and follow-up long-term chronic disease management by General Practitioners (GPs) are both accessible and utilized. Being decentralized also enables individual care nodes to operate efficiently according to their operational requirements while retrofitting their protocols to the wider PHLE ecosystem.

#### Health triggers

Health triggers refer to predefined clinical, behavioral, or contextual indicators that automatically prompt preventive or follow-up actions within a health system. These may include biometric thresholds (e.g., elevated blood pressure), missed appointments, or changes in risk profiles detected through digital monitoring (16). Health triggers translate latent risk into scheduled demand and, hence, into anticipatory demand by applying queuing theory, particularly Little's Law, which links arrival rates, service times, and work-in-process. Clearly defined triggers for action stabilize otherwise volatile demand patterns, especially among high-risk populations. By smoothing patient flow, these mechanisms reduce backlog and waiting times while enabling timely intervention on upstream determinants of health, ultimately improving both efficiency and equity in preventive care delivery. Keying in the required clinical and paraclinical parameters will initiate workflows, including next-episode screening, referral to higher levels of care, or social prescribing activities. At the population level, mobile applications can also be synchronized with health status consolidated across various care interfaces and trigger alerts for future healthcare visits, lifestyle events, or changes in blood investigation results.

#### Service flows

Service flows in the PHLE utilize the SCOR model to manage preventive pathways as an integrated, end-to-end chain, rather than fragmented steps. This approach prevents bottlenecks from merely shifting between silos, increases completion rates across the continuum of care, and safeguards scarce capacity by ensuring that each stage of the preventive process is coordinated and aligned. This should be assisted by an interoperable IT system that integrates basic health information across all care interfaces, so that a lack of patient information does not inhibit prompt service delivery and enables providers to know which services are required for the patient.

#### Integration layer

The integration layer provides the structural backbone of the PHLE, drawing on Donabedian's model. A shared logistics platform enables continuity of care across multiple actors by pooling resources, standardizing workflows, and aligning incentives. Embedding logistics functions directly into regional health planning ensures that operational considerations are treated as core components of preventive strategies rather than afterthoughts, thereby promoting both feasibility and equity in delivery. An integrated layer that enables services required and delivered at all care nodes can also promote transparency and meet agreed service obligations. Having a bird's-eye view of all logistical needs and their respective deliverables can also open the door to efficient policy and systems evaluation.

#### Metrics for system performance

PHLE evaluation links operational design to outcomes also using Donabedian's model while aligning with the SCOR framework's performance categories for comparability and control. The proposed metrics extend beyond clinical results to capture logistical effectiveness, including equity of access across socioeconomic groups, time-to-care from risk identification to intervention, and reductions in avoidable hospitalizations. By making the operational “health” of preventive care visible, these indicators provide policymakers and system leaders with actionable insights for continuous improvement and accountability.

## Applying PHLE in Healthier SG: policy and system implications

Singapore's HSG strategy offers a timely test case for how preventive health reform can be translated from policy into practice. Yet without a robust logistical backbone, even well-designed strategies risk remaining aspirational. Preventive services in Singapore continue to be predominantly anchored within clinic- and hospital-based settings, with persistent disparities in the geographical accessibility of CHAS-accredited general practitioners across housing estates (17). Among older adults, mobility constraints, limited digital literacy, and reliance on caregivers further impede engagement with preventive care, while fragmentation across service providers (GPs, community partners, and hospital outreach teams) creates duplication and inefficiencies.

The framework explicitly addresses several gaps that existing solutions have struggled to resolve. First, clearly defined health-trigger systems can reduce missed opportunities for screening and follow-up, especially among older adults with fluctuating attendance patterns. Second, decentralized care nodes mitigate mobility and digital-access barriers by bringing services closer to neighborhoods where older residents live. Third, the integration layer helps prevent duplication (such as repeated assessment by different agencies) by enabling shared scheduling, information flows, and logistics coordination. Collectively, these mechanisms provide actionable pathways for strengthening the last-mile delivery of preventive services.

Elements of the PHLE, however, are already visible within Singapore's health system. The introduction of multidisciplinary “Healthier SG Teams” (18) embodies the decentralized care node principle by linking clinical and community actors at the precinct level. Likewise, Delivery on Target Primary Care Networks (DOT PCN) (19) demonstrate how independent GP practices can be networked to pool resources, standardize workflows, and coordinate chronic-disease management. The Agency for Integrated Care (AIC) has further advanced integration by designating lead social service agencies to coordinate health and social services within regions, aligning incentives and logistics across sectors. Together, these initiatives illustrate the early application of PHLE principles in operational settings.

To achieve scale, Healthier SG must now consolidate these efforts through a deliberate logistics infrastructure. A shared integration layer anchoring scheduling, information flows, and service obligations across care nodes would ensure that bottlenecks are managed system-wide rather than shifted between silos. Financing mechanisms should explicitly recognize the logistical work inherent in prevention, including mobile deployment, coordination time, and transport operations, rather than remunerating only clinic encounters. Digital solutions such as HealthHub (20) can function as population-level health-trigger systems, but must be complemented by analog pathways for digitally marginalized seniors.

Anchored in PHLE principles, we identify the following five recommendations.

### Embedding logistics expertise

Preventive health planning should explicitly integrate logistics and operations specialists alongside clinicians and policymakers. Professionals experienced in supply chain management, flow design, and last-mile distribution (traditionally confined to hospital procurement) can enhance demand forecasting, capacity planning across care nodes, and coordinate service flows in primary and community care. Embedding such expertise within Ministry of Health transformation teams, Regional Health Offices, and Healthier SG implementation units would ensure interventions are designed with operational feasibility from the outset.

### Sustained resource allocation

Decentralized nodes and mobile models, such as neighborhood health pods and mobile outreach units, require dedicated and recurrent funding to sustain operations. *Ad-hoc* or pilot-based funding cycles undermine scalability. Financing frameworks should treat the logistics infrastructure (such as vehicles, coordination teams, community spaces, and scheduling systems) as long-term system investments, comparable to hospitals or polyclinics.

### Accountability through system performance metrics

Performance management must evolve beyond clinical outcomes to place greater emphasis on accountability through logistics-related system metrics and Key Performance Indicators (KPIs). These should encompass time-to-care (from risk identification to intervention), equity of access across socioeconomic groups, and the avoidance of duplication across providers. Such metrics make the operational “health” of preventive systems visible, enabling policymakers to detect bottlenecks early and drive continuous improvement.

### Person-centered design

Preventive logistics must reflect the lived realities of seniors and caregivers. Co-designing service flows—such as offering accessible venues, flexible appointment timing, and analog alternatives to digital enrolment (21)—enhances participation and sustainability. Integrating caregiver engagement and feedback loops ensures logistical efficiency translates into meaningful user experience, promoting equity and trust.

### Aligning financing with logistics

Sustainable implementation depends on aligning payment models with the logistical realities of prevention. Under capitation or risk-pooling arrangements, activities such as transport, coordination, and mobile deployment should be recognized as cost-saving investments. Funding should follow patients across settings, supported by outcome-based payments tied to successful navigation of high-risk individuals through the preventive pathway.

By embedding these enablers, HSG could evolve from an enrolment-driven strategy into a dependable, neighborhood-based delivery model. Strengthening the logistics infrastructure (alongside clinical, digital, and financial reforms) would operationalise the PHLE framework in practice, ensuring that preventive care becomes accessible, coordinated, and sustainably delivered for Singapore's aging population.

## Conclusion

The challenges observed in Singapore's experience with preventive health reform are mirrored across aging societies such as China, Japan, South Korea, and much of Europe (22, 23), where over-reliance on facility-based delivery, fragmented provider networks, and digital or social exclusion persist as barriers to equitable care. As population's age and health systems pivot toward prevention, *how* services are delivered will become as critical as *what* is delivered.

The PHLE framework offers a transferable response. By conceptualizing prevention as a logistics ecosystem, it provides adaptable principles for diverse contexts. Decentralized care nodes may take the form of rural outreach teams in China (24) or municipality-led community centers in Europe (25), but the underlying logic (bringing preventive services closer to populations) remains constant. Similarly, health triggers, service flows, integration platforms, and performance metrics can be adapted to varying governance and resource environments while maintaining system coherence. Rather than prescribing a fixed model, the PHLE serves as a scalable architecture that health systems can selectively layer to align with existing strategies in digital health, primary care, and social services, thereby strengthening operational readiness for population aging.

Ultimately, the PHLE reframes preventive care as an operational discipline rather than a purely policy or behavioral endeavor. Embedding logistics science into system design links preventive strategies to measurable flows, performance indicators, and resource allocation, transforming intent into implementation. Logistics should therefore be recognized not as an auxiliary support, but as a foundational pillar of preventive health reform, on par with clinical quality and financial stewardship. The experience of HSG and comparable initiatives globally demonstrates that success hinges on integrating logistical planning across policy, financing, and operational domains. Doing so will enable health systems to move from aspiration to execution, fostering resilience and ensuring that preventive care remains accessible, coordinated, and people-centered.
